# Regional assessment of choroidal vascularity index in patients with pre- and early-stage diabetic retinopathy using ultra-wide-field OCTA

**DOI:** 10.3389/fmed.2024.1490831

**Published:** 2024-10-24

**Authors:** Yulei Chen, Haoxiong Xian, Minghui Liu, Xiuqing Dong, Shaolin Du

**Affiliations:** ^1^Department of Ophthalmology, Dongguan Tungwah Hospital, Dongguan, China; ^2^Dongguan Key Laboratory of Eye and Systemic Diseases, Dongguan, China; ^3^First Clinical Medical College, Guangdong Medical University, Zhanjiang, China

**Keywords:** diabetes mellitus, diabetic retinopathy, optical coherence tomographic angiography, choroidal vascularity index, renal function

## Abstract

**Purpose:**

To characterize the regional variations of choroidal vascularity index (CVI) in patients with diabetes mellitus (DM) using ultra-wide-field optical coherence tomography angiography (UWF-OCTA) and identify their correlations with the onset of diabetic retinopathy (DR).

**Methods:**

This cross-sectional, monocular-sampling study recruited 141 participants from four age-matched groups: no DM (NDM), no DR with early DM (EDM) and late DM (LDM), and mild–moderate non-proliferative diabetic retinopathy (mNPDR). UWF-OCTA was employed for circular scans centered on the fovea. CVI in the central region (0–1 mm) and four quadrants of the concentric rings with different ranges (1–3, 3–6, 6–9, 9–12, 12–15, 15–18 mm) was obtained for analysis together with their demographic and clinical data. The Area under the receiver operating characteristic curve (AUC) was calculated to assess the diagnostic efficacy for mNPDR and compared using the DeLong test.

**Results:**

The average CVI was lower in patients with mNPDR compared to other groups across all regions. Although there was no significant difference in DM duration between the LDM and mNPDR groups, a notable variance in CVI was observed, particularly (*p* = 0.0004) in the temporal quadrant of the 15–18 mm range (T18). CVI in T18 region was negatively correlated with creatinine levels, while positively correlated with body mass index and estimated glomerular filtration rate (*p*s < 0.05). The CVI in the T18 region demonstrated superior diagnostic efficacy (AUC = 0.755), and when combined with those in other regions and clinical data, the AUC rose to 0.907, which was significantly better (*p* = 0.0280) than using clinical data alone.

**Conclusion:**

Reduced CVI was observed in the most peripheral region, highly predictive for mNPDR and associated with the declining renal function, thus enhancing the potential of UWF-OCTA to integrate into DM management and promote early DR screening.

## 1 Introduction

Diabetic retinopathy (DR) is one of the most common microvascular complications in patients with diabetes mellitus (DM), and the leading cause of irreversible visual impairment among the population of working age (1). With the increasing prevalence of DM estimated to implicate 700 million individuals globally by 2045 (2), the DR screening is required to escalate concomitantly. DR’s progression is typically a multi-year journey, starting from mild to moderate non-proliferative diabetic retinopathy (mNPDR), advancing to macular edema or/and proliferative diabetic retinopathy. Notably, mNPDR is the initial phase of DR encompassing the majority of DR patients (3). Mild-to-moderate non-proliferative retinopathy (mNPDR) is characterized by microaneurysms, retinal hemorrhages, ischemia, cotton wool spots, hard exudates, intraretinal microvascular abnormalities and venular dilation and tortuosity (4). However, challenges remain in screening mNPDR due to the absence of ocular symptoms and the minimal lesion such as retinal microaneurysm, requiring prolonged follow-up of DM patients and the time-consuming fundus examination by experienced ophthalmologists. Early diagnosis and intervention of mNPDR are desirable to provide new insights into DM management and prevent the vision loss secondary to DR. Consequently, it is necessary to develop diagnostic indicators with non-invasive and convenient features to predict and monitor the onset of DR.

Choroid vascular changes are presumed to offer a window into DR predictors. While choroid vessels provide about 90% of the blood flow for the eye, especially for the outer retina including photoreceptors and the retinal pigment epithelium, diabetes may lead to choroidal abnormalities similar to those observed in the retina, such as microaneurysms, vascular tortuosity, vascular outpouchings, areas of vascular non-perfusion and neovascularization, which are considered characteristic features of diabetic choroidopathy and can occur along with the onset of DR (5–8). Jankowska-Lech et al. (9) observed the hypofluorescent and hyperfluorescent area in choroid at various stages of DR using indocyanine green angiography (ICGA) in cases with no apparent abnormalities under fundus fluorescein angiography (FFA), which suggested that choroidopathy precedes retinopathy in DR patients. Compared to ICGA and FFA, optical coherence tomographic angiography (OCTA) is characterized by high-speed and non-contact quantitative imaging, making it a promising modality for the clinical detection and assessment of diabetic choroidopathy (10, 11).

Derived from OCTA, the choroidal thickness and choroidal vascularity index (CVI) was documented to enable quantitative analysis of choroidal vascular changes. Current evidence suggested that CVI, which corresponds to the ratio of luminal area to total choroidal area, allows for a more stable and objective assessment of the structural changes in choroidal circulation abnormalities (12–14). Most studies confirm the decreasing CVI along with the severity of DR but lack agreement on mNPDR (15, 16). Given that the conventional OCTA had a field of view (FOV) within 6 × 6 mm (17–19), peripheral CVI has not been adequately studied yet. Recently, some studies characterized the peripheral CVI changes in patients with early-stage DR using wide-field OCTA (WF-OCTA) with the FOV enlarged to 12 × 12 mm (20, 21). However, current studies based on WF-OCTA are inconsistent and questionable in detecting early-stage DR, probably due to the considerable variability in DM duration between groups of the study subjects and the lack of independence stemming from binocular sampling. As the importance of an expanded FOV has been illustrated by the ultra-wide-field (UWF) imaging in color fundus photography and fluorescein angiography for early detection of several chorioretinal diseases, the need to bring similar utility to OCTA is imperative for screening DR (22, 23). Qing Zhao et al. (24) also employed ultra-wide-field OCTA (UWF-OCTA) to assess central and peripheral changes in the retina and choroid in DM patients without clinical DR. The potential of UWF-OCTA and CVI in the field of DR screening remains to be developed. Moreover, previous studies mainly assessed the relationships between CVI, and blood glucose associated indices, whereas the link between the renal function and CVI, especially in a larger FOV, has not been fully investigated.

In this study, we utilized relatively novel UWF-OCTA (25, 26) to achieve an 18-mm diameter quantitative imaging for the CVI from the choroidal central to peripheral regions in the DM patients. We aimed to investigate the regional variation of CVI and assess its correlations with mNPDR and clinical indices to clarify the value of UWF-OCTA in predicting and monitoring the onset of DR.

## 2 Materials and methods

This cross-sectional observational study was approved by the Institutional Review Board of Dongguan Tungwah Hospital (DHKY-2024-016-01) and conducted in accordance with the Declaration of Helsinki. 141 eyes of 141 individuals referred from endocrinology and ophthalmology departments, identified with random number generation method (27) for monocular sampling, were enrolled in this study with written informed consent. Randomization was based on the random number generator function in Excel (Microsoft Office 365; Microsoft, WA), which can generate a random integer between 0 and 1. If the number is 0, the left eye was sampled; if it is 1, the right eye was sampled. And their demographic and clinical data were obtained retrospectively from electronic medical records within a week before the enrollment.

Participants with or without DM, diagnosed according to the diagnostic criteria of the American Diabetes Association (28) or the self-reported history of diabetes mellitus, were recruited at Dongguan Tungwah Hospital in Dongguan, China from October 2023 to April 2024. Ophthalmic examinations, including intraocular pressure (IOP) measurement (NT-510, Nidek, Japan), slit lamp fundus examination (YZ5X, 66 Vision Tech, China), and scanning laser ophthalmoscopy (Daytona PT200, Optos, United Kingdom), was carried out on the same day in each participant. Several exclusions were then applied as follows: (1) evidence or history of any clinically visible ocular diseases (except for dry eye, non-pathological myopia and mild cataract) such as glaucoma, retinal detachment, and macular edema; (2) any recent ocular topical medications experience (within 1 month) including dry eye-associated medications; (3) history of previous ocular surgery such as focal laser, retinal photocoagulation and intravitreal injections; (4) any OCTA image with quality index less than 18 or presence of substantial motion artifacts or center shifts; (5) IOP exceeding 21 mm Hg. Level of DR was defined according to the International Classification of Diabetic Retinopathy (29), and the duration of DM was defined as the interval from the date of self-reported initial diagnosis to the date of the eye examination for each participant in this study. Only DM patients with mNPDR and without diabetic macular edema in the fundus photographs, identified by a trained ophthalmologist, were included in the mNPDR group, while age-matched individuals without DR were further subdivided according to their DM duration into following groups: the healthy controls without DM (no DM, NDM), the patients with short duration of DM ≤ 1 years (early DM, EDM) and with the long duration of DM > 1 years (late DM, LDM).

**Figure 1:**
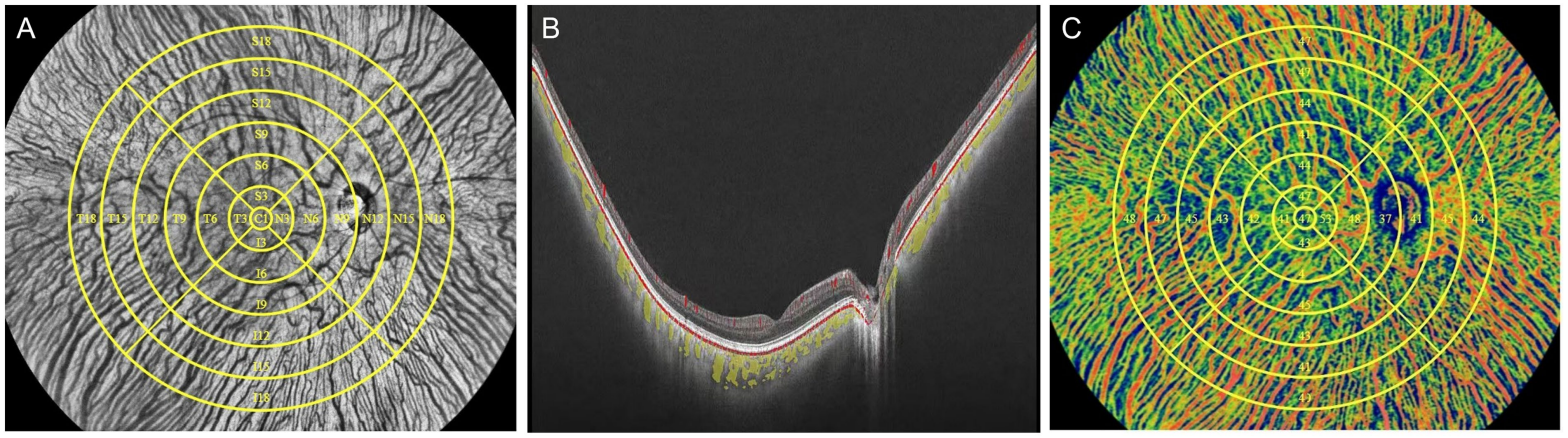
Schematic illustration of processing to obtain the CVI using UWF-OCTA. (A) In face structural OCT image of the fundus with 25 subregions segmented manually in yellow. The largest circle determined the 18-mm diameter circular region that was separated into the central circular subregion with a diameter of 1 mm (C1 region) and the concentric rings with diameters of 1–3 mm, 3–6 mm, 6–9 mm, 9–12 mm, 12–15 mm, and 15–18 mm, and further divided into four quadrants: superior (S), inferior (I), nasal (N), and temporal (T). Each subregion is labeled with the corresponding alphanumeric name. (B) B-scan image of the fundus with the lumen of the large and medium choroidal vessels pseudo-colored as yellow. (C) Choroidal OCTA image in the pseudo color mode with regional CVI measurements indicated in the corresponding region.

Each participant underwent imaging by UWF-OCTA in a dark room between 9 a.m. and 5 p.m. and meditated for 10 min before the examination to minimize daily variation in choroidal perfusion. The UWF-OCTA was a swept-source OCTA system (BM-400K, TowardPi Medical Technology Co., Ltd., Beijing, China) using a swept-source laser with a central wavelength of 1,060 nm and a scan rate of 400,000 A-scans per second (25, 26). The resolution in the axial direction and transversal direction was 3.8 μm and 10 μm, respectively, with the scan depth of 6 mm. A FOV of 120 degrees was selected for 24 × 20 mm B-scans centered on the fovea. As shown in Figure 1A, the 18-mm diameter circular area was abstracted from 24 × 20 mm OCTA image and divided into total 25 regions including the central region (0–1 mm) and 4 quadrants of the concentric rings with different ranges (1–3, 3–6, 6–9, 9–12, 12–15, 15–18 mm). The choroid was stratified into different sub-layers as follows: the inner capillary layer, the Sattler’s layer of medium vessels in the middle, and the outer Haller’s layer with large vessels, by a built-in custom segmentation software with occasional manual correction (Figure 1B). The CVI, defined as the ratio of luminal area to total choroidal area, was calculated for each region across the Sattler’s layer and Haller’s layer using the typical binarization method (30) by the built-in software (Figure 1C).

All statistical analysis was performed using GraphPad Prism software, version 9.0.0 and MedCalc version 20.0.4 (MedCalc Software, Ostend, Belgium). Data were tested for normality using Shapiro–Wilk normality test and subsequently assessed for homogeneity of variance by the Bartlett’s test. Categorical variables were shown as proportions, whereas continuous variables were presented as mean ± standard deviation. Categorical variables among groups between subgroups were compared by the Chi-squared test. Difference of numerical variables including regional CVI among groups was calculated by ordinary one-way analysis of variance (ANOVA) with Tukey–Kramer multiple comparisons test (for data that passed normality tests) or Kruskal-Wallis test with Dunn’s multiple comparisons test (for data that did not pass normality tests), while difference of CVI among regions within group was calculated by Friedman test. Correlations between variables were evaluated with the Pearson correlation tests. Receiver operating characteristics (ROC) analysis was performed to determine the diagnostic efficacy for mNPDR in DM patients and areas under the ROC curve (AUC) were calculated. Difference of AUCs was tested using a DeLong test. For all statistical tests, *p* < 0.05 was considered statistically significant.

## 3 Results

### 3.1 Demographic and clinical characteristics

**Figure 2:**
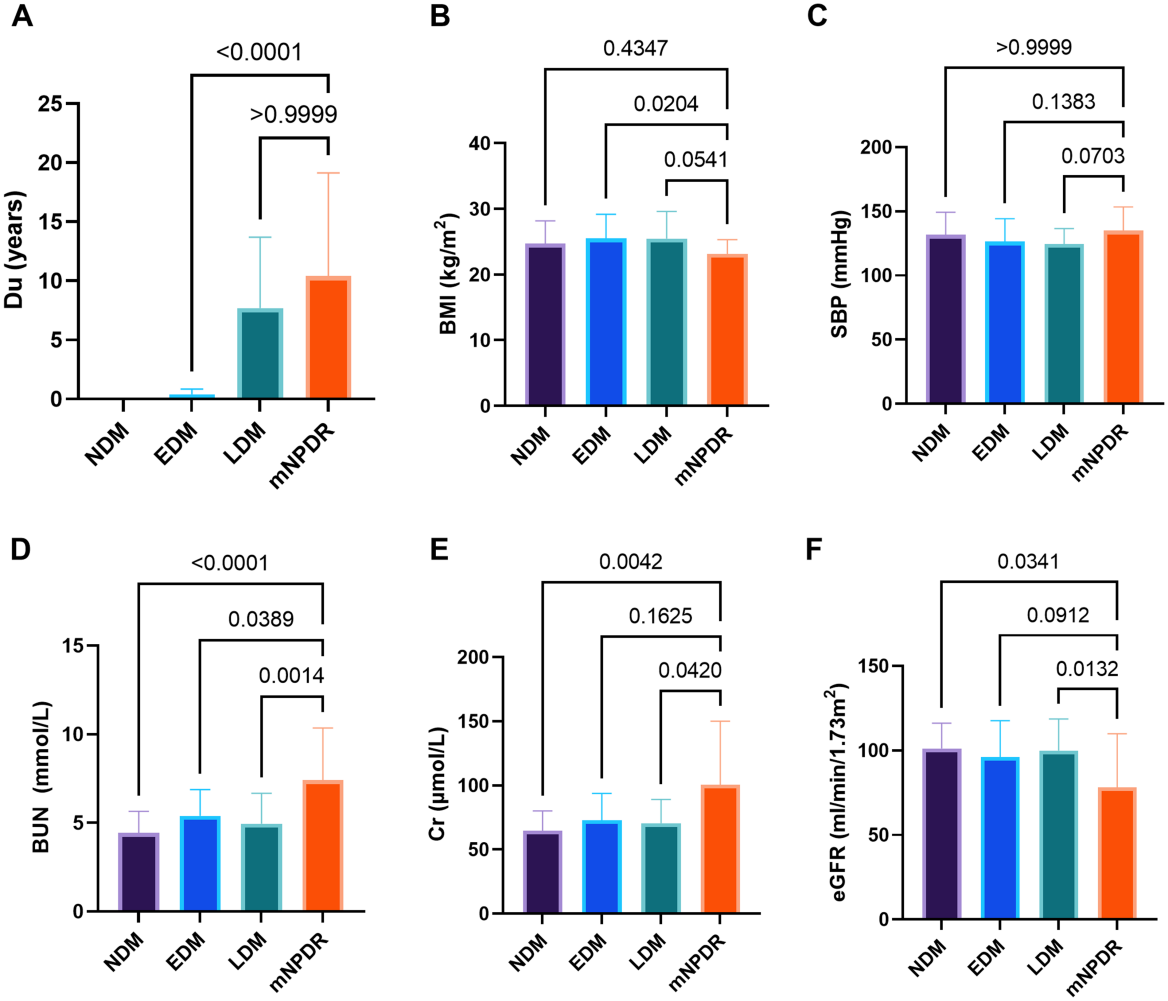
Comparison among groups in terms of duration (A), BMI (B), SBP (C), BUN (D), Cr (E), and eGFR (F). *p*-values noted on the corresponding position for between-group comparisons, were calculated using Tukey–Kramer multiple comparisons test or Dunn’s multiple comparisons test. Statistically significant values are shown as *p* < 0.05 marked by*, *p* < 0.01 marked by**, *p* < 0.001 marked by***, *p* < 0.0001 marked by****. Du, diabetes duration; BMI, body mass index; SBP, systolic blood pressure; BUN, blood urea nitrogen; Cr, blood Creatinine; eGFR, estimated glomerular filtrate rate; NDM, the controls with no DM; EDM (early DM), the patients with short duration of DM ≤ 1 years; LDM (late DM), the patients with the long duration of DM > 1 years; mNPDR, the patients with mild–moderate non-proliferative diabetic retinopathy; ns, non-significant.

**Figure 3:**
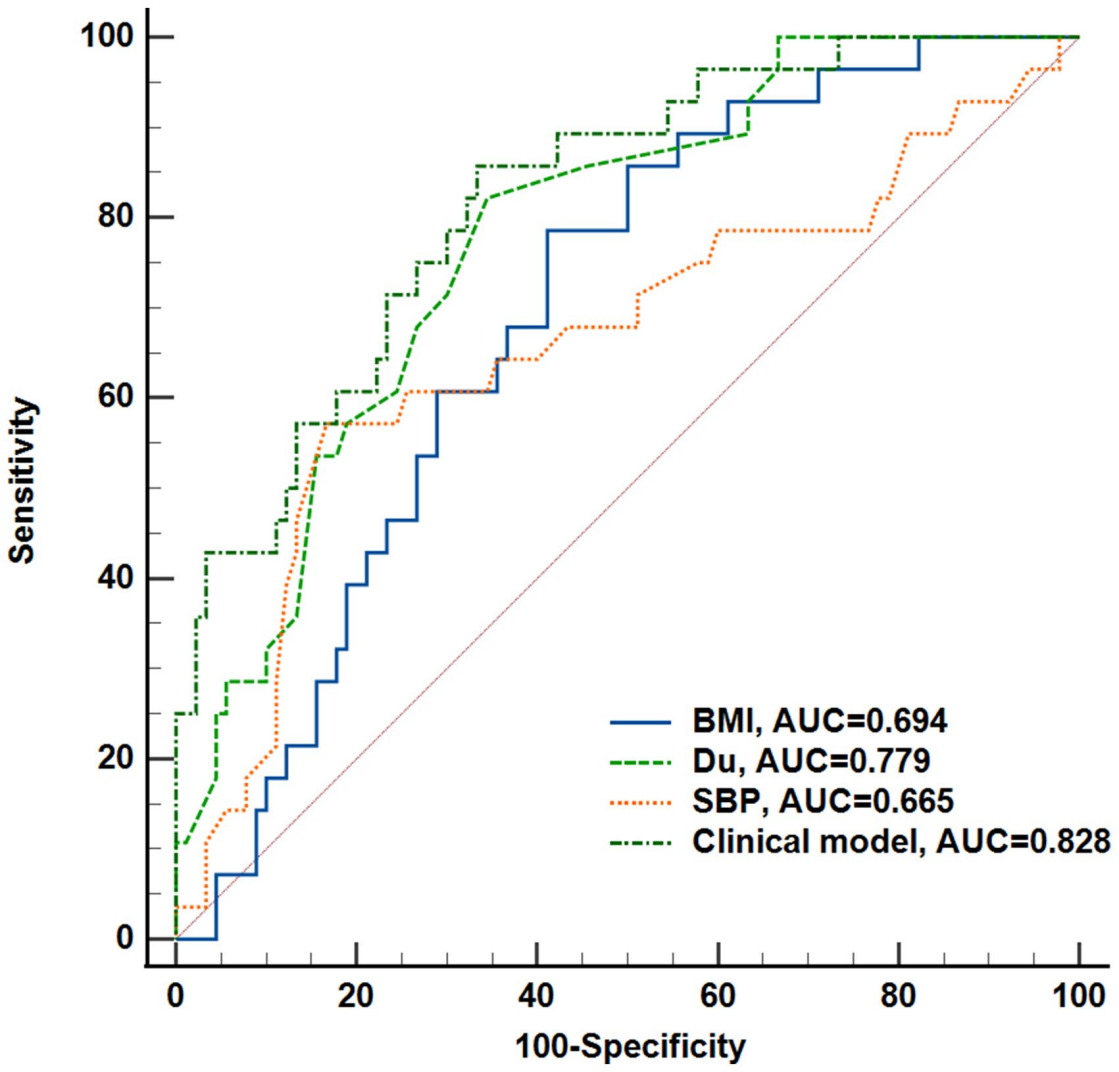
Performance of the non-invasive clinical indicators (BMI, Du, SBP, Weight) and their combination (clinical model) to predict DR onset. Receiver operating characteristic curves and Delong’s test was used to compare the diagnostic efficacy. Du, diabetes duration; BMI, body mass index; SBP, systolic blood pressure.

A total of 141 eyes from 141 individuals enrolled in this study, was consisted of four groups whose demographic and clinical features were shown in Table 1. All groups were matched for age, gender and the ratio of right to left eyes. Among the four groups, significant differences were found in the term of BMI, duration of DM (Du), SBP, BUN, Cr and eGFR (*p*s < 0.05) but not in HbA1c, SBP, TC, TG or LDL (*p*s > 0.05). The most striking difference among groups was observed for Du and BUN (*p*s < 0.0001) among the groups. These indices indicating significant differences were subsequently selected for further comparative analyses as shown in Figure 2. The mean duration of LDM and mNPDR were 7.69 ± 6.01 and 10.41 ± 8.72 years, respectively, with no significant difference (*p* > 0.9999). BMI and SBP did not differ between LDM and mNPDR (*p* = 0.0541, *p* = 0.0703, respectively). BUN, Cr and eGFR showed significant differences between LDM and mNPDR but did not show significant differences among the NDM, EDM and LDM groups (*p* > 0.05). Compared to the LDM groups, mNPDR groups exhibited significant differences as increase in Du, BUN and Cr, and decrease in eGFR. As shown in Figure 3, Comparisons of ROC curves indicated the higher diagnostic power of Du for mNPDR compared to BMI and SBP, although no significant difference among AUCs of these three non-invasive clinical indicators were confirmed by Delong test (Du vs. BMI, *p* = 0.2240; Du vs. SBP, *p* = 0.1872; BMI vs. SBP, *p* = 0.7505). And the combination of them (named the clinical model) presented the better diagnostic capacity which is significantly higher than that of BMI and SBP (Clinical model vs. BMI, *p* = 0.0103; Clinical model vs. SBP, *p* = 0.0136; Clinical model vs. Du, *p* = 0.308).

**Table 1:** Demographic and clinical characteristics of the non-diabetic subjects and DM patients.

Characteristics	NDM (*n* = 23)	DM without DR		mNPDR (*n* = 28)	*p* values
		EDM (*n* = 49)	LDM (*n* = 41)		
Gender (*n*, female/male)	14/9	18/31	14/28	7/21	0.0560
Enrolled eyes (*n*, OD/OS)	9/14	25/24	20/22	16/12	0.6267
Age (years)	50.13 ± 10.31	48.67 ± 10.5	51.18 ± 11.16	53.86 ± 11.33	0.3280
Duration of DM (years)	0	0.4 ± 0.44	7.69 ± 6.01	10.41 ± 8.72	<0.0001****
Height (m)	1.62 ± 0.09	1.65 ± 0.09	1.66 ± 0.08	1.66 ± 0.09	0.2420
Weight (kg)	64.63 ± 10.24	71.22 ± 14.81	68.44 ± 11.64	63.14 ± 9.45	0.0502
BMI (kg/m^2^)	24.73 ± 3.46	25.98 ± 4.18	24.82 ± 3.42	23.16 ± 2.17	0.0219*
HbA1c (%)	N/A	10.17 ± 2.4	9.28 ± 2.14	8.7 ± 2.18	0.0870
SBP (mmHg)	131.78 ± 17.46	126.98 ± 15.56	123.45 ± 15.37	134.89 ± 18.49	0.0413*
DBP (mmHg)	77.57 ± 13.09	79.61 ± 9.42	74.38 ± 11.32	80.21 ± 7.55	0.3502
TC (mmol/L)	4.76 ± 1.00	4.95 ± 1.38	5.24 ± 1.88	4.52 ± 1.18	0.4439
TG (mmol/L)	2.14 ± 2.75	3.59 ± 5.96	2.84 ± 3.23	2.04 ± 1.54	0.1553
LDL (mmol/L)	2.87 ± 0.93	3.01 ± 1.00	3.07 ± 1.31	2.72 ± 1.13	0.5362
BUN (mmol/L)	4.45 ± 1.2	5.07 ± 1.54	5.38 ± 1.67	7.42 ± 2.93	<0.0001****
Cr (μmol/L)	64.83 ± 15.34	70.11 ± 19.67	74.02 ± 19.41	100.63 ± 49.5	0.0053**
eGFR (ml/min/1.73 m^2^)	101.2 ± 15.04	99.77 ± 20.33	95.28 ± 20.18	78.23 ± 31.69	0.0099**

Data were presented as the means ± standard deviations for the four groups: NDM, the control group with no DM; EDM, the patients with short duration of DM; LDM, the patients with long duration of DM; mNPDR, the DM patients with mNPDR and without DME. Difference of numerical variables among groups was assessed using one-way ANOVA or Kruskal-Wallis test, while Chi-square test was performed for categorical variables. Significant differences are shown as *p* < 0.05 marked by*, *p* < 0.01 marked by**, *p* < 0.001 marked by***, *p* < 0.0001 marked by****.

BMI, body mass index; OD, right eyes; OS, left eyes; SBP, systolic blood pressure; DBP, diastolic blood pressure; HbA1c, glycosylated hemoglobin; TC, total cholesterol; LDL, low-density lipoprotein; TG, triglyceride; BUN, blood urea nitrogen; Cr, blood Creatinine; eGFR, estimated glomerular filtrate rate; N/A, not applicable.

### 3.2 Regional CVI variation

**Figure 4:**
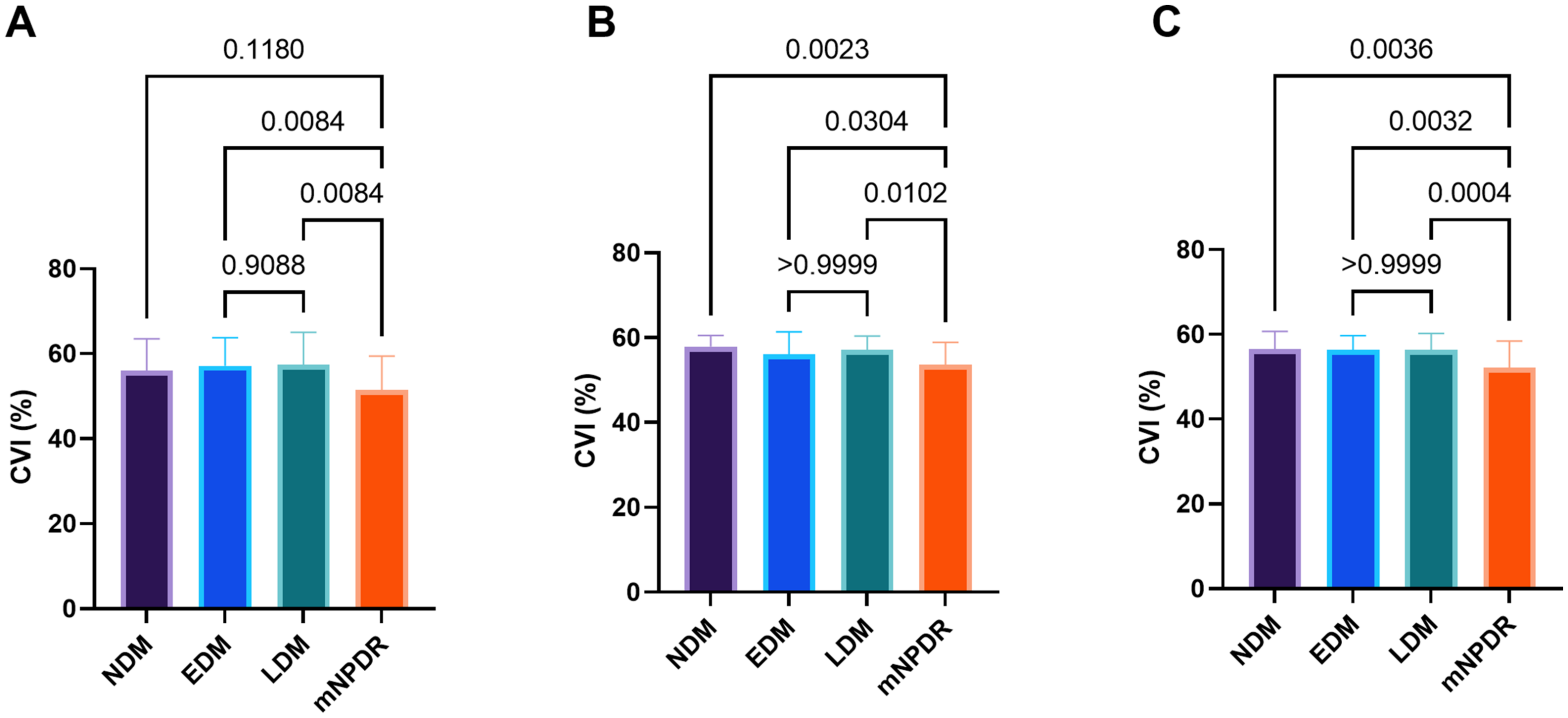
Comparison among the four groups in terms of CVI in C1 **(A)**, N15 **(B)** and T18 **(C)** regions, respectively. Differences were examined by Tukey–Kramer multiple comparisons test or Dunn’s multiple comparisons test. p values were noted on the corresponding position for between-group comparisons. Statistically significant values are defined as *p* < 0.05.

**Figure 5:**
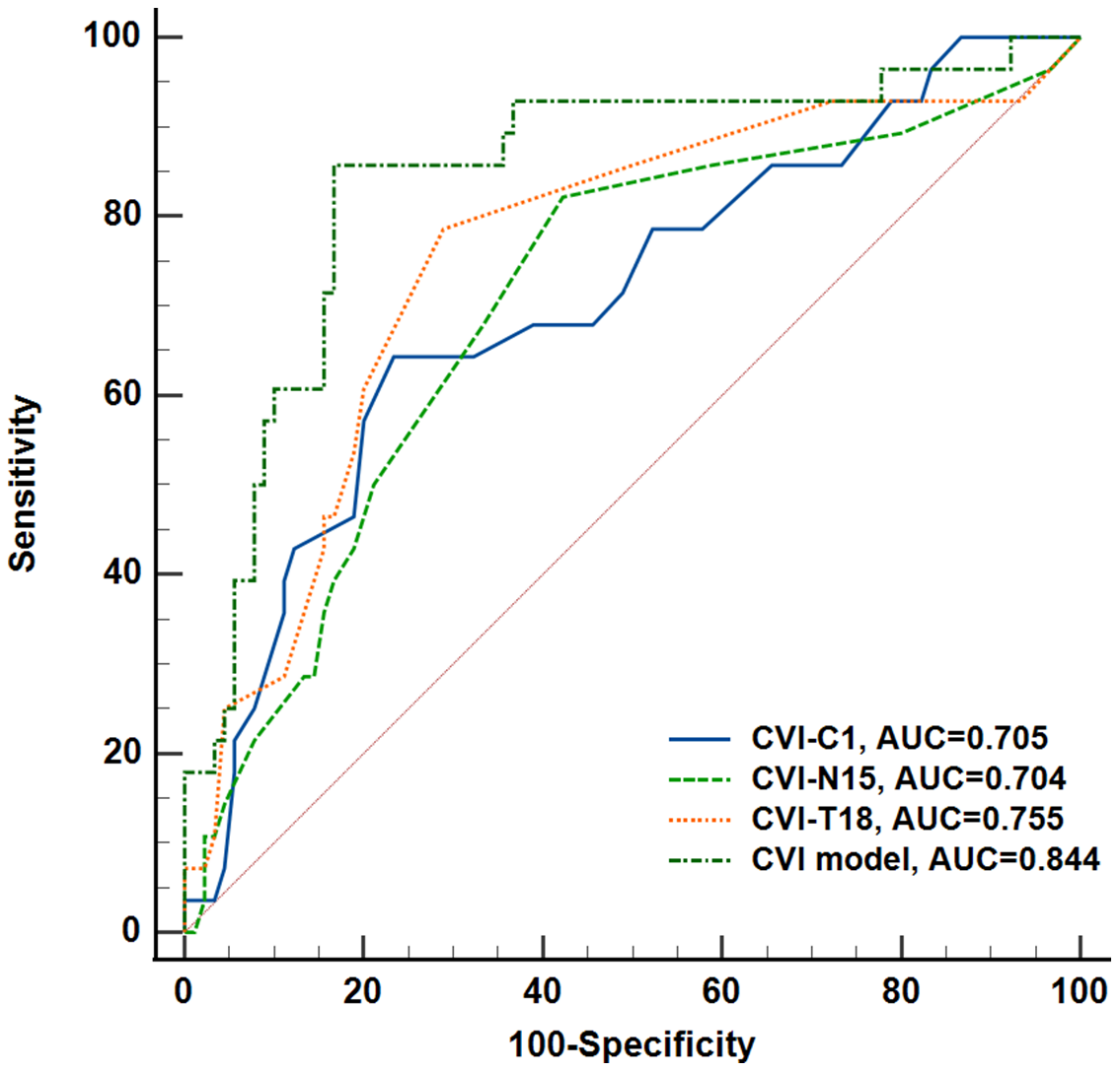
Performance of CVI in C1, N15, T18 regions, and the combination of all regions (named CVI model) to predict DR onset. Receiver operating characteristic curves and Delong’s test was used to compare the diagnostic efficacy.

The regional CVI measurement results of each group were shown in Table 2. The Friedman test demonstrated that there were significant differences across regions within each group (*p*s < 0.05). And there were particularly significant differences (*p*s < 0.001) observed among groups in N15 (*p* = 0.0018) and T18 (*p* = 0.0003) regions. *Post hoc* comparisons among groups in N15 and T18 regions, as well as the subfoveal choroidal region (C1 region), are presented in Figure 4. The CVI in C1, N15 and T18 regions was significantly lower in patients with mNPDR than in the EDM and LDM groups, while there were no significant differences (*p*s > 0.05) between the EDM and LDM groups. The most notable variance between LDM and mNPDR groups was observed (*p* = 0.0004) in T18 region. Subsequently, the performance of these three regions and the combination of total 25 regions (named CVI model) in mNPDR prediction was depicted in Figure 5. The AUCs of CVI showed 0.705 (95% CI: 0.614–0.785), 0.704 (95% CI: 0.613–0.784), 0.755 (95% CI: 0.667–0.829) and 0.844 (95% CI: 0.766–0.904) for C1, N15 and T18 regions and the CVI model, respectively. While no significant difference among them were confirmed by Delong test (C1 vs. N15, *p* = 0.9875; C1 vs. T18, *p* = 0.4307; N15 vs. T18, *p* = 0.1576), the CVI model presented the better diagnostic capacity which is significantly higher than that of C1 and N15 (CVI model vs. C1, *p* = 0.0236; CVI model vs. N15, *p* = 0.0414; CVI model vs. T18, *p* = 0.1489).

**Table 2:** Comparisons of regional CVI values within and among groups.

Regions	NDM	DM without DR		mNPDR	*p* values
		EDM	LDM		
Circle 0–1 mm					
C1	56.00 ± 7.51	57.16 ± 6.58	57.62 ± 7.80	51.50 ± 7.91	0.0060**
Circle 1–3 mm					
N3	55.96 ± 4.82	55.06 ± 4.97	55.20 ± 4.76	52.61 ± 6.52	0.3869
S3	56.17 ± 5.09	55.00 ± 5.65	56.88 ± 5.30	52.43 ± 5.97	0.0077**
T3	56.48 ± 4.73	55.76 ± 5.06	55.76 ± 4.87	52.36 ± 5.80	0.0547
I3	55.83 ± 3.88	55.92 ± 4.79	55.76 ± 4.77	52.71 ± 6.79	0.2400
Circle 3–6 mm					
N6	56.17 ± 3.13	56.16 ± 4.66	56.48 ± 4.16	52.18 ± 5.68	0.0041**
S6	56.22 ± 2.68	55.67 ± 3.73	55.81 ± 3.70	52.82 ± 6.01	0.0175*
T6	56.52 ± 4.96	56.71 ± 3.91	56.60 ± 4.17	53.29 ± 5.94	0.0278*
I6	56.43 ± 3.29	56.08 ± 4.48	56.69 ± 4.13	52.96 ± 5.22	0.0036**
Circle 6–9 mm					
N9	55.48 ± 3.99	52.47 ± 7.63	54.95 ± 5.07	51.25 ± 7.16	0.0256*
S9	56.52 ± 2.48	55.59 ± 3.59	56.31 ± 3.47	53.29 ± 5.62	0.0268*
T9	54.17 ± 7.63	52.96 ± 5.43	54.50 ± 5.43	50.79 ± 6.81	0.0259*
I9	57.17 ± 2.76	55.39 ± 4.37	56.55 ± 3.28	53.04 ± 5.29	0.0041**
Circle 9–12 mm					
N12	55.78 ± 4.31	53.06 ± 6.81	55.55 ± 4.42	52.46 ± 5.76	0.0302*
S12	56.39 ± 2.71	55.94 ± 3.58	56.38 ± 3.15	53.68 ± 5.03	0.0819
T12	54.39 ± 6.41	53.59 ± 5.86	54.00 ± 5.41	50.07 ± 8.10	0.0371*
I12	57.09 ± 2.95	56.12 ± 4.31	57.19 ± 3.22	53.46 ± 7.01	0.0218*
Circle 12–15 mm					
N15	57.91 ± 2.57	56.02 ± 5.36	57.10 ± 3.23	53.61 ± 5.31	0.0018**
S15	57.09 ± 2.59	56.10 ± 3.55	56.83 ± 3.00	53.79 ± 4.97	0.0065**
T15	56.83 ± 4.39	55.98 ± 4.41	57.07 ± 4.02	51.86 ± 7.29	0.0024**
I15	56.61 ± 3.93	55.88 ± 4.18	56.50 ± 3.46	52.39 ± 7.46	0.0117*
Circle 15–18 mm					
N18	57.48 ± 2.39	55.43 ± 6.16	57.10 ± 2.79	53.46 ± 5.49	0.0022**
S18	57.17 ± 2.67	55.94 ± 3.31	56.62 ± 3.19	53.61 ± 5.50	0.0043**
T18	56.52 ± 4.20	56.31 ± 3.37	56.45 ± 3.81	52.14 ± 6.26	0.0003***
I18	55.91 ± 4.50	55.59 ± 3.75	56.12 ± 3.56	52.50 ± 5.09	0.0026**
*p* values	0.0031**	<0.0001****	<0.0001****	0.0047**	

Regional CVI values were presented as the means ± standard deviations for the four groups. *p* values in the last row were indicated for within-group comparisons and calculated by Friedman test, while *p* values in the last columns were indicated for intergroup comparisons and calculated by one-way ANOVA or Kruskal-Wallis test. Statistically significant values are shown as *p* < 0.05 marked by*, *p* < 0.01 marked by**, *p* < 0.001 marked by***, *p* < 0.0001 marked by****.

DM, diabetic mellitus; DR, diabetic retinopathy; NDM, the controls with no DM; EDM (early DM), the patients with short duration of DM ≤ 1 years; LDM (late DM), the patients with the long duration of DM > 1 years; mNPDR, the patients with mild–moderate non-proliferative diabetic retinopathy.

### 3.3 Correlation between CVI and clinical indices

**Figure 6:**
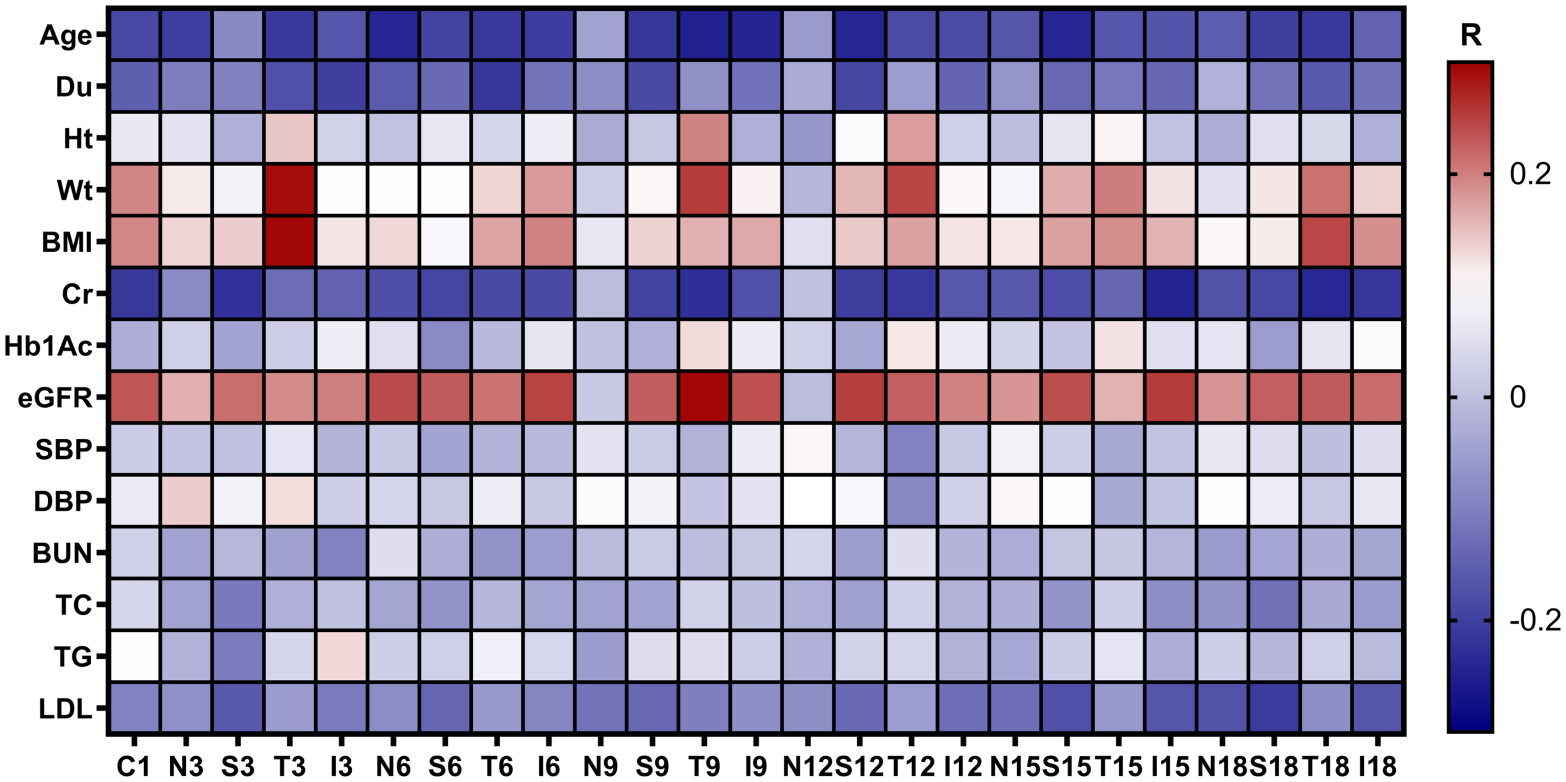
Correlation between regional CVI values with demographic and clinical characteristics. The *R* values are the Pearson correlation coefficients colored with a blue to red heatmap. Du, duration of diabetes mellitus; Ht, height; Wt, weight; BMI, body mass index; Cr, blood Creatinine; HbA1c, glycosylated hemoglobin; eGFR, estimated glomerular filtrate rate; SBP, systolic blood pressure; DBP, diastolic blood pressure; BUN, blood urea nitrogen; TC, total cholesterol; TG, triglyceride; LDL, low-density lipoprotein; N/A, not applicable.

As shown in Figure 6, the Pearson correlation tests revealed that in patients with DM including the NDM, LDM and mNPDR groups, the correlation between CVI in most of regions and eGFR was weakly positive, yet significant (*p* < 0.05, *R* < 0.3). There are also weakly negative but significant correlations of CVI in most regions with age and Cr. Of the cases, significant correlations were found between CVI in T18 region and age (*p* = 0.02, *R* = −0.21), Cr (*p* < 0.01, *R* = −0.24) and eGFR (*p* < 0.01, *R* = 0.23). In addition, decreasing CVI values were associated with declined BMI (*p* < 0.01, *R* = 0.24) as observed in T18 region.

**Figure 7:**
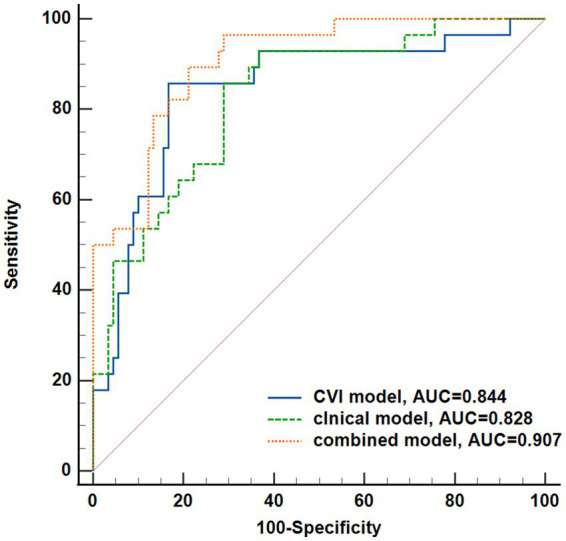
Performance of the three model to predict DR onset. CVI model, the combination of CVI from each region; Clinical model, the combination of the non-invasive clinical indicators (BMI, Du, SBP); Combined model, the combination of the previous two models. Difference of receiver operating characteristics among the three models was tested using a DeLong test.

The performance of the CVI model and clinical model as well as the combination of these two (named combined model) in DR onset prediction was compared as shown in Figure 7. The combined model resulted in AUC of 0.909 (95% CI: 0.842–0.954), showing the ability to distinguish the mNPDR eyes in patients which was significantly higher than that of the CVI model and clinical model (Combined model vs. CVI model, *p* = 0.0222; Combined model vs. Clinical model, *p* = 0.0280; CVI model vs. Clinical model, *p* = 0.7214).

## 4 Discussion

The choroid is a highly vascularized tissue comprised of five layers, three of which are vascular: the inner capillary layer, Sattler’s layer of medium vessels in the middle, and the outer Haller’s layer with large vessels (24). Choroidal changes in each vascular layer due to hyperglycemia in diabetes can be quantified by CVI more stably and robustly than by the choroidal thickness (25). CVI was stable across various choroidal thicknesses, and symmetrical between the eyes of an individual. Since the traditional OCTA was limited by imaging depth and width, most previous experimental studies of CVI have focused on choriocapillaris and subfoveal area, indicating that CVI tended to decline along with the worsening DR but remained inconsistency on mNPDR. Valencia Hui Xian Foo et al. (26) studied the CVI at the 3 × 3 mm macular region and demonstrated that the alteration seemed to be located at the Haller’s layer and Sattler’s layer as the DM developed and progressed. Until WF-OCTA enlarged to a 12 × 12 mm scanning area, Fabao Xu et al. (16) and Zhihao Qi et al. (15) reported that the changes of CVI were more significant in the peripheral choroid between the DM patients without DR and those with DR. Notably, these studies involved binocular sampling in most of the participants with a lack of independence between samples, thereby might lead to a wrong statistical inference, over- or underestimating the correlation between CVI and the development of DR. Recently, Ç. Keskin et al. reported that the subfoveal CVI tended be lower in diabetic patients with NPDR compared to the non-DR by performing comparison among groups with considerable variation of DM duration (13). Given that mNPDR can occur in the different stage of DM, the diagnostic performance of CVI for mNPDR especially in the patients with similar duration of DM wasn’t shed light in the above studies. Therefore, in this cross-sectional study based on monocular sampling of each participant, UWF-OCTA examinations were performed on four groups (healthy subjects, patients with new-onset DM, patients with long-standing DM, and DM patients with mNPDR) and the imaging area was enlarged to 18-mm diameter with refinement of regional assessment covering medium- and large-sized vessels in the choroid.

The reliability of UWF-OCTA for CVI was first demonstrated in the assessment of the normal eyes. The average value of subfoveal CVI was 56% ± 7.51% (ranged from 42 to 70%) in agreement with previous reports (27–29). Subsequent Friedman test showed that there were significant differences in variance (*p* < 0.05) noted among CVI measured from all assessed regions for these healthy subjects without DM. These findings diverged from prior research using conventional OCTA with a limited range of measures, showing that single regional measurement of choroidal vascularity could not represent total choroidal vascularity in healthy population. Remarkably, the distribution of CVI was not uniform among different regions both for new-onset and long-standing DM patients (*p*s < 0.05). Presumably this means that there was a reflection of changes in regional variance dissimilar to total changes. More refined regional assessment of CVI may contribute to examining more sensitive measures of choroidal vascular changes. Thus, total 25 choroidal regions, more than that of previous studies, were designed to be evaluated separately by UWF-OCTA in this study. Nevertheless, it’s noted that the comparisons among the controls, EDM and LDM groups indicated no significant changes in CVI, no matter which choroidal regions was tested (*p*s > 0.05). Even the subfoveal CVI exhibited a rising trend as the duration of diabetes increased although without statistical significance, as described in Figure 4. This might be occasioned by a compensating mechanism for decreased vascular blood perfusion in retinal and choroid capillaries of diabetic patients. Moreover, the CVI value in other regions was almost concordant between the controls without DM and DM patients whatever with short- or long-duration disease (*p*s > 0.05). This result differs from previous study on choriocapillaris and supports the previous literature reports on Sattler’s and Haller’s layer, inferring that DM did not have a large enough impact on medium-large vessels preceding the onset of DR.

By contrast, the CVI was significantly lower in patients with mNPDR than in control subjects or DM-NoDR patients for most of regions, especially for N15 and T18 regions. Comparison between the LDM and mNPDR groups revealed similar DM duration but significant differences in CVI for these regions. C1, the subfoveal choroidal region, has been a typical focus in previous studies. Consequently, we included C1 in our analysis alongside the N15, and T18 regions. Of these, C1 region showed the largest mean difference but without the maximum significance in CVI between the LDM and mNPDR groups. Significant difference in CVI between the controls and mNPDR groups was noted for the N15 and T18 regions but not for C1 region. This observation indicates that choroidal vascular changes may already exist in this central area of DM-NoDR eyes and resulted in the escalated CVI for C1 region although not significantly, which was so less stable that its discriminative capacity for mNPDR is instead reduced. In comparison, the CVI for the N6, T15 and T18 regions appeared relatively stable in the progression of DM until the occurrence of mNPDR. The difference due to mNPDR was most significant for the T18 region. As we surmised, ROC analysis yielded an average AUC of 0.755 for the T18 region, which had the best diagnostic accuracy among all the regions and reached a higher diagnostic efficacy (AUC = 0.909) when integrating those in other regions and the invasive clinical data (Figure 7). This observation highlighted the importance of regional vascular changes in the peripheral choroid for predicting the onset of DR and the highly promise of UWF-OCTA used for the early diagnosis of DR.

To consolidate the potential of the CVI from UWF-OCTA, we further analyzed their relationships with demographic and clinical data obtained from the patients’ electronic medical record. Our results verified that CVI for almost all regions were unrelated to DM duration, blood pressure, HbA1c and blood lipids level. This can possibly be used to explain the previous reports that long disease duration, hyperglycaemia, hypertension, and high lipids, although recognized as major risk factors, lack the predictive power for development and progression of DR. The duration of diabetes is indeed a commonly used indicator for predicting diabetic retinopathy as reported by current studies but not significantly correlated with CVI in this study. While there were no significant differences in diabetes duration between the LDM and mNPDR groups, our result suggested that there were notable differences between the two groups in term of CVI. The performance of the CVI model exerted the superior diagnostic efficacy with a satisfactory sensitivity and specificity, and when used in combination with those non-invasive clinical indicators (Du, BMI and SBP), the diagnostic power was improved significantly (AUC = 0.909, *p* = 0.022). In this regard, it was proved that the combination of regional assessment of CVI with other clinical indicators could improve the diagnostic power, which may provide new ideas for clinical practice. It is also noted that CVI significantly correlated with BMI (*p* = 0.008), as did the measures of renal function including Cr (*p* = 0.009) and eGFR (*p* = 0.01). Hence, we can reason that the patients with weight loss or/and renal function decline deserves more attention and intervention to prevent DR. Additionally, some recent studies have begun to explore these kidney functional parameters, explaining the tight correspondence between the choroidal changes and chronic kidney disease. More recently, Wenbo Zhang et al. (30) reported the decrease in the CVI parallels the severity of kidney disease. In this sense, UWF-OCTA has promising clinical applications to afford more sensitive assays as a non-invasive diagnostic indicators and monitoring biomarker of retinopathy and nephropathy.

Furthermore, we believe these findings should be supported by future studies with larger population and longer follow-up period throughout the progression of DR. Diet and medication administered during DM management may have an impact on choroidal vascular flow and physiological indices, thus should be assessed independently to explored new insight into early intervention at the early stage of DR. Since this study reported on DM patients without distinguishing kidney disease, which can have significant effects on kidney function, a more detailed classification of diabetic nephropathy should be established to address the limitations of this study.

In conclusion, this study presents the regional assessment of CVI via UWF-OCTA in patients with mNPDR, enabling the detection of regional vascular changes in the peripheral choroid and strengthening the capacity for monitoring the onset of DR. Further, we verified the decreased CVI as a function of renal function in DM patients. With non-invasive and highly sensitive properties, UWF-OCTA appears to be a promising measure to integrate into clinical management of DM and promote the early diagnosis of DR.

## Data Availability

The original contributions presented in the study are included in the article/supplementary material, further inquiries can be directed to the corresponding author.
